# An Exercise Health Simulation Method Based on Integrated Human Thermophysiological Model

**DOI:** 10.1155/2017/9073706

**Published:** 2017-06-15

**Authors:** Nan Jia, Xiaohui Chen, Liang Yu, Ruomei Wang, Kaixing Yang, Xiaonan Luo

**Affiliations:** ^1^The School of Data and Computer Science, Sun Yat-sen University, Guangzhou 510006, China; ^2^School of Computer and Information Security, Guilin University of Electronic Technology, Guilin 541004, China

## Abstract

Research of healthy exercise has garnered a keen research for the past few years. It is known that participation in a regular exercise program can help improve various aspects of cardiovascular function and reduce the risk of suffering from illness. But some exercise accidents like dehydration, exertional heatstroke, and even sudden death need to be brought to attention. If these exercise accidents can be analyzed and predicted before they happened, it will be beneficial to alleviate or avoid disease or mortality. To achieve this objective, an exercise health simulation approach is proposed, in which an integrated human thermophysiological model consisting of human thermal regulation model and a nonlinear heart rate regulation model is reported. The human thermoregulatory mechanism as well as the heart rate response mechanism during exercise can be simulated. On the basis of the simulated physiological indicators, a fuzzy finite state machine is constructed to obtain the possible health transition sequence and predict the exercise health status. The experiment results show that our integrated exercise thermophysiological model can numerically simulate the thermal and physiological processes of the human body during exercise and the predicted exercise health transition sequence from finite state machine can be used in healthcare.

## 1. Introduction

There is evidence that healthy exercise can minimize the physiological effects of an otherwise sedentary lifestyle and increase active life expectancy by limiting the development and progression of chronic disease and disabling conditions [1]. Research on healthy exercise is important and has been focused on for the past few years.

During exercise, the human body exchanges energy with the clothing systems and environmental conditions in different forms of heat transfer; a coupled system about thermoregulatory mechanism is determined based on the Human-Clothing-Environment (HCE) [2, 3]. Particularly, the thermoregulatory responses of the body and the sensory responses of skin nerve endings follow the laws of physiology [4]. The human active tissues produce additional metabolic heat, which must be intricately offset by heat loss to the environment [2, 4]. The core temperature increases and several physiological reactions in internal temperature regulating system are automatically activated to accelerate body heat dissipation including sweating by stimulating the sweat gland and automatically adjusting the cardiovascular system [5]. During cardiovascular adjustment, the blood is redistributed from the core organs to the skin to facilitate heat dissipation, and the active muscles require blood supply to deliver oxygen for maintenance of activities. The heart rate increases correspondingly to sustain cardiac output and blood supply to the working muscles and the skin [6].

With the dynamic changes of physiological indicators during exercise, many health phenomena such as thirst, breathing disorders, and dizziness can appear. Without adopting effective preventive measures in time, health accidents (dehydration, exertional heatstroke, syncope, and even sudden death) may happen [4]. At the Standard Chartered Hong Kong Marathon 2013, 55 runners were reported to have fallen unconscious, been rendered comatose, and suffered from collapse because of heatstroke; more than 100 athletes have died from excessive heat stress because of exertional heat stroke during competitions in the recent 20 years [6]. If these health accidents can be analyzed or predicted before they happened, it will be beneficial to alleviate or avoid disease and mortality [7]. Hence, the research of exercise physiological performance is significant for the health monitoring, analysis, and accident precaution.

Some technologies have been used to obtain the body physiological performances and predict the health states. The wearable health monitoring system (WHMS) usually takes the advanced sensory technology to get the immediate physiological values and then deal with these values for real-time health judgment and risk prediction [8, 9]. For example, a large variety of laboratory prototypes, test beds, and industrial products of WHMS [10, 11] have already been produced. The Nike+ Fuel Band is an activity tracker worn on the wrist to track wearers' physical activity, heart rate, and amount of energy burned [12]. The My Heart project [13] and the SmartVest project [11] are smart clothes, where the sensing modules are either garment-integrated or simply embedded on the piece of clothing. All of them need participants to put on various wearable products at any moment to collect continuous physiological data. They are costly and inconvenient for daily exercise sometimes. Data mining (DM) method takes advantage of the historical exercise data and personal health data to assess or predict the health status [14]. Various data mining methods have been adopted to deal with physiological information and predict the human health status. Li and Clifford applied a multilayer perceptron neural network to estimate the quality of the pulses in PPG [15]. Pantelopoulos and Bourbakis presented a health prognosis methodology based on fuzzy regular language [16]. Calderon and de Brito introduced data mining models such as decision tree, *k*-nearest neighbors (*k*NN), and support vector machine (SVM), for analyzing electrocardiograms (ECG) in order to identify heart attack and the probability of incidence [17]. But, if there is no enough historical physiological data for a participant to analyze, the accuracy of prediction may be a big problem.

Computer simulation modeling in exercise healthcare is an attractive proposition. Obtaining the mathematical model that describes the human physiological regulation mechanisms can improve our understanding of exercise physiology and is helpful for the prediction of health accidents during exercise. Some significant research results can be developed around human thermal behavior simulation as well as the human heart rate response simulation. Reviewed by Cheng et al. [18, 19], all the models for human body can be characterized in terms of their viewpoints of development. They are (1) one-node model [20], (2) two-node model [21], (3) multinode model [22–24], and (4) multielement model [25, 26]. All these models can simulate the thermal performance of human body, while their mechanisms such as heat conduction, sweating, vasoconstriction, and vasodilatation can be implemented from simple to complex. In the one-node model, human body is regarded as a single node, and it is only applicable to thermal environment. In the two-node model, human body is divided into core and skin; the basic thermoregulation mechanisms such as heat conduction, sweating, vasoconstriction, and vasodilatation can be simulated. This model is easy to be understood and implemented. In the multinode and multielement models, the division of the human body is customized to the requirement of researchers. In these two models, a series of complex mathematical equations is used to describe the more physiological mechanisms (e.g., the blood perfusion phenomenon, the negative feedback control process). These two models require complex simulation settings and higher computation abilities, and they can obtain local physiological performance. Physiological models about cardiovascular system in human body increasingly receive attention in recent years. Cheng et al. proposed a series of nonlinear heart rate models to simulate the heart rate regulation process during exercise [27, 28]. Ataee et al. developed a low-order lumped parameter model to describe the autonomic-cardiac regulation behaviors [29]. Buller et al. presented a quadratic regression model to implement heart rate regulation by controlling the human core temperature [30, 31].

From the literature review performed above, we have found that the fundamental knowledge of human thermoregulation mechanisms has been established. Several physiological indexes can be numerically computed by the mathematical model. However, the existing models focus on different emphasis points; the thermal performances and human physiological performances are simulated individually. The relationship between these performances has not been established in the existing work. Besides, some problems such as what method can be used to predict the exercise healthy status and how to alleviate or avoid exercise accidents before they happen are unresolved. Therefore, it is important to develop a comprehensive simulation model integrating various human regulation mechanisms to obtain human thermal performance and physiological performance during exercise. Further, based on these exercise simulation results, some research on healthy exercise is conducted.

In this study, we propose an integrated human exercise physiological model, in which a two-node human thermal physiological model and a nonlinear heart rate response model are coupled together to simulate the human physiological regulatory mechanism; a series of thermal and physiological performances can be computed according to the numerical computation model. Both human thermal sensation (temperature of skin, relative humidity of skin) and physiological status (core temperature, sweat rate, skin blood flow, and heart rate) are obtained. They are important in understanding, analyzing, predicting, and preventing the health problems (accident, disease, etc.) during exercise. Then, a fuzzy logical method is employed to deal with our simulated results in exercise health prediction. Specifically, a special fuzzy finite state machine is defined to describe the health state transition in exercise process. Finally, two different cases are designed to evaluate the proposed approach. Compared with the existing approaches, our approach can be used to predict the health status before the exercise starts. Further, the research results can be used in the healthcare service which may also be beneficial in predicting and reducing cardiovascular disease mortality [32]. This may also lead to an improvement in developing training protocols for athletes and more efficient weight loss protocols for the obese and in facilitating evaluation of physical fitness and health of individuals [33]. To clarify, we noted the importance of computer simulation technique in the study of human sports and proposed a method to assess human exercise comfort in 2016 [34]. Different from the work of this paper, the previous one employs human physiological model to obtain physiological indicators and defines a set of fuzzy rules to measure the human comfort, while this work applies the obtained physiological indicators as input of a complicated fuzzy finite state machine and then quantifies the human exercise health status.

## 2. Method

**Figure 1:**
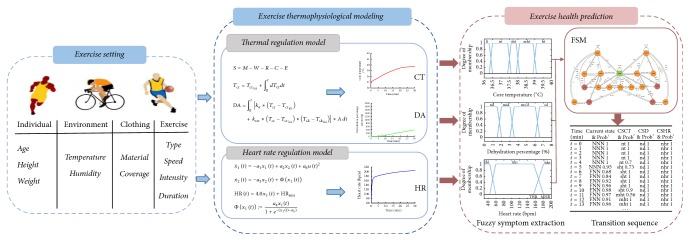
Flow chart of health simulation approach.

Figure 1 shows the flow chart of the exercise health simulation method, in which the various parameters in the left side are input and the predicted health state list is output. From Figure 1 we can see the important issues are exercise thermophysiological modeling and exercise health prediction. In exercise thermophysiological modeling, two important simulation models must be considered, which are human thermal regulation model and heart rate regulation model. Using the simulated results, an exercise health prediction model is developed. That can be used in the exerciser to obtain the healthy exercise effects.

### 2.1. An Integrated Exercise Thermophysiological Simulation Model

During exercise, active tissues in body will produce large amount of heat; this will break the body thermal balance and affect the human physiological performances. Hence, human body thermophysiological regulation mechanisms used to speed up body heat dissipation are activated to make the body in a proper thermal status. Such regulation mechanisms mainly include sweating by stimulating the sweat gland and automatically adjusting the cardiovascular system. Modeling of these regulation mechanisms (especially the thermoregulatory mechanism and heart rate regulation mechanism) is significant. According to the literature review we can find that the heat and moisture performances of human body can be simulated by some mathematical models. The basic thermoregulation data such as heat conduction, sweating, vasoconstriction, and vasodilatation, as well as the physiological indicators of human core temperature, human skin temperature, sweat rate, and so on, can be simulated. On the other hand, the individual physiological regulation models are presented. In this paper, a nonlinear heat rate regulation model is integrated into the human heat and moisture transfer model to simulate the exercise thermophysiological performances. Compared with the thermal simulation model, the main character of the integrated simulation model is focusing the human exercise thermophysiological properties. Particularly the human heart rate can be simulated during exercise.

#### 2.1.1. Two-Node Thermal Regulation Model

Considering the complexity and efficiency of numerical simulation, a two-node thermal regulation model is used to simulate the thermal behaviors and represent the thermoregulatory mechanisms of the human body [21]. Core temperature and dehydration amount are main parameters used in the exercise health prediction model. Some mathematical equations are used to calculate these two parameters.

The mathematical equation of two-node thermal regulatory model in unit skin area is presented as follows:

(1) $$\begin{array}{c} S=M-W-R-C-E, \end{array}$$

where *S* is the rate of heat storage, *M* is the rate of metabolic heat production, *W* is the heat loss by exercise accomplished, *R* is the heat gained or lost by radiation, *C* is the heat gained or lost by convection, and *E* is the total evaporative heat loss, and it includes the heat of vaporized moisture from the lungs during respiration (*E*_res_), the heat of vaporized water diffusing through the skin layer (*E*_diff_), and the heat of vaporized sweat necessary for the regulation of body temperature (*E*_rsw_). It should be noted that there is a positive correlation between *M* and exercise intensity. Therefore, when exercise intensity increases, the rate of metabolic heat production increases.

In detail, the mathematical models can be represented as follows:

(2) S=Ssk+Scr,Ssk=Kmin∗Tcr−Tsk+cbl∗Vbl∗Tcr−Tsk−R+C,Scr=M−Eres−W−Kmin∗Tcr−Tsk−cbl∗Vbl∗Tcr−Tsk,

where *S*_sk_ is the rate of heat storage in core, *S*_cr_ is the rate of heat storage in core, *T*_sk_ is the skin temperature, *T*_cr_ is the core temperature, *K*_min_ is the minimum heat conductance of skin tissue, *c*_bl_ is the specific heat of blood, and *V*_bl_ is the rate of skin blood flow.

With the heat storage changed, the values of skin and core temperature at any simulation time can be calculated as follows:

(3) Tsk=Tskini+∫0tSsk∗Amsk∗cskdt,Tcr=Tcrini+∫0tScr∗Amcr∗ccrdt,

where *dT*_sk_ is the skin temperature change rate, *dT*_cr_ is the core temperature change rate, *T*_sk_ini__ is the initial temperature of skin, *T*_cr_ini__ is the initial temperature of core, *m*_cr_ is the core mass, *c*_cr_ is the core specific heat capacity, and *A* is the body surface area, it is a function of body height and weight proposed by Schlich et al. [35].

Sweating is usually caused by temperature stimuli from both the skin and core. An effective sweating mechanism can take away the additional heat and help human body work well during exercise. The sweat rate *m*_rsw_ is used to measure the performance of the sweating mechanism in our model, and it is written as follows:

(4) mrsw=ka∗Tcr−Tcrini+ksw∗Tcr−Tcrini∗Tsk−Tskini∗A,

where *T*_sk_ini__ and *T*_cr_ini__ are the initial values of *T*_sk_ and *T*_sk_, *T*_sk_ − *T*_sk_ini__ and *T*_cr_ − *T*_cr_ini__ can be seen as the temperature control signals (they are responsible for the thermoregulatory control actions), *k*_*a*_ is the coefficient of the additional sweat amount during activities, and *k*_sw_ is the coefficient of sweating rate model.

The sweating accumulation in (5) can be used to diagnose whether the body is dehydrated or not; it is defined as dehydration amount (DA) in this paper.

(5) $$\begin{array}{c} DA=\overset{t}{\underset{0}{\int }}m_{rsw}dt. \end{array}$$

#### 2.1.2. Heart Rate Regulation Model

Heart rate regulation behaviors are important in maintaining a physiological balance state in exercise process. During exercise, large amount of blood is required to facilitate heat dissipation and deliver oxygen into muscles. The human heart rate increases to sustain cardiac output and blood supply to the working muscles and the skin. Nonlinear heart rate regulation model aiming to simulate the heart rate behaviors and represent the heart rate regulation mechanisms of the body can be introduced [27]. In this model, the neuroregulation mechanism can well reflect the dramatic change of heart rate especially in the strenuous exercise. The thermal regulation mechanism combined with some other mechanisms is usually utilized to describe the slow-acting effects of HR. The mathematical equations of the nonlinear heart rate regulation model are presented as follows:

(6) x˙1t=−a1x1t+a2x2t+a6ut2,x˙2t=−a3x2t+Φx1t,HRt=4.0∗x1t+HRrest,Φx1t≔a4x1t1+e−x1t−a5,

where *x*_1_(*t*) describes the change of HR mainly due to the neural effects to exercise (the effects comprise the sympathetic and parasympathetic), *x*_2_(*t*) describes the change of HR due to the peripheral effects comprising the human thermoregulation system, the hormonal system, and other physiological phenomena, *u* is the exercise intensity, and it directly affects human metabolic rate, *a*_*i*_  (*i* = 1,…, 6) is positive parameter which depends on the specific individual performing various exercise, HR_rest_ is the heart rate at rest, and its default value is 74, and HR is the output we need.

### 2.2. Exercise Health Prediction

Applying various indicators obtained from the proposed thermophysiological model to predict the potential exercise health risk is a worthwhile method. Among the various simulated physiological indicators, core temperature, dehydration amount, and heart rate are the most important ones in exercise symptoms diagnosing, and they are chosen as health prediction variables.

#### 2.2.1. Fuzzification

**Figure 2:**
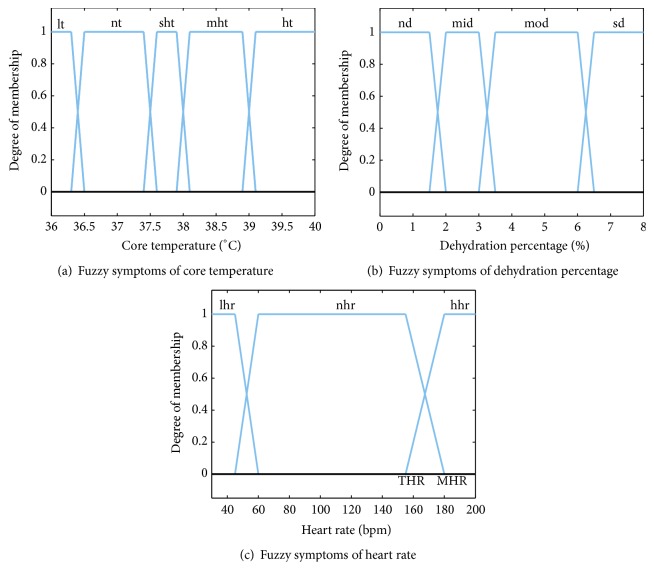
Fuzzy symptoms extracted from simulated indicators.

Instead of characterizing simulated physiological indicators in a crisp manner, we can employ fuzzy logic [36] to describe the degree of occurrence of a certain indicator. Particularly, the trapezoidal function in fuzzy logic is selected to define the membership function of every input indicator. With the guidance of medicine experts, the severity interval for health symptoms is divided and the corresponding fuzzy symptoms are obtained.  Figure 2 shows fuzzy symptoms extracted from simulated indicators. Especially in the heart rate membership function, the THR is the target heart rate, which indicates the recommended optimal heart rate [37]. MHR is the maximum heart rate that the human body can tolerate [38]. These two thresholds are directly related to the participant's age and exercise intensity; their values should be calculated as follows:

(7) MHR=163+1.16∗age−0.018∗age2,THR=MHR−HRrest∗EIP+HRrest,

where HR_rest_ is the rest heart rate (its default value is 74) and EIP is the exercise intensity percentage.

As shown in Figure 2, concerning the core temperature, the human health is commonly classified into three states, that is, hypothermia, normothermia, and hyperthermia. The hypothermia usually shows symptom of low temperature (lt). The normothermia shows symptom of normal temperature (nt). The symptoms of hyperthermia include slightly high temperature (sht), moderate high temperature (mht), and high temperature (ht). The dehydration amount is classified into two states, that is, normal and dehydration. The normal shows symptom of nondehydration (nd). The dehydration shows three symptoms, namely, mild dehydration (mid), moderate dehydration (mod), and severe dehydration (sd). The heart rate is also classified into three states, that is, bradycardia, normal, and tachycardia. Each state corresponds to one symptom, namely, low heart rate (lhr), normal heart rate (mhr), and high heart rate (hhr), respectively.

#### 2.2.2. Finite State Machine Definition

Once the fuzzy symptoms are generated, we need to predict the health transition states based on the obtained fuzzy data. Traditional rule-based health judgment method is wildly used to calculate the health state of discrete time [39], the degree of healthy, and the health tendency during the whole process which are unknown. Therefore, the finite state machine (FSM) [40] is introduced and applied in exercise health prediction. Finite state machine is useful in the situations where behavior is driven by many different types of events; the response to a particular event depends on the sequence of previous events. In this case, the change of CT, DA, and HR can be used as trigger events and a specific finite state machine is defined to simulate the health transition sequence during exercise. The FSM is represented as a 4-tuple (Σ, *Q*, *ϕ*, *δ*), where we have the following:

1. ****Σ denotes the set of all possible health symptoms extracted from the simulated physiological data. The total number of symptoms in the current FSM is 12 (5 + 4 + 3). All the symptoms and their corresponding notations are listed in Table 1. Each symptom has a degree of membership (DOM) 0 ≤ *μ*(*i*, *j*, *x*) ≤ 1, which denotes the certainty or strength of the corresponding symptom, where *i* belongs to the set of all indicators simulated by our physiological model, *j* belongs to the set of all symptoms that can be extracted from the *i*th indictor, and *x* is the simulated value. For example, *μ*(1,3, 37.7) means that the current core temperature is 37.7°C, and the symptom of core temperature is slightly high temperature. The current membership degree of *μ*(1,3, 37.7) is 1.
2. *Q* denotes the set of all possible health states. These states signify the various possible combinations of health symptoms presented in Σ. The total number of health states in the current FSM is 18 (3*∗*2*∗*3). These health states and their possible syndromes [41, 42] are summarized in Table 2, where the first letter signifies the state of the core temperature, the second letter means the percentage of dehydration amount, and the third letter means the heart rate. The state NNN is usually regarded as the beginning state; any state can be regarded as the final state when the exercise ends.
3. *ω* denotes the weighting function. It associates a weight with every transition rule in the FSM and represents the causal associations between symptoms and unhealthy/healthy states. This function is commonly based on the medicine knowledge [41] and it is helpful to determine the occurrence of a health state. In current FSM, all the transitions weight values are set equal to 1; for example, for HR signs, *ω*_*N*→*N*_ = *ω*_*N*→*T*_ = *ω*_*N*→*B*_ = *ω*_*T*→*T*_ = *ω*_*B*→*B*_ = *ω*_*B*→*N*_ = 1.
4. *δ* denotes the transition function. The state transition in FSM is in the form of* A*→*αB*, where A (e.g., NNN) signifies the current health state,* B* (e.g., NNT) is the new estimated health state (*B* can be equal to* A*), and *α* (e.g., hhr) is a new extracted symptom being processed. That is, by accepting a new symptom of hhr, the state NNN can be changed to NNT.

**Figure 3:**
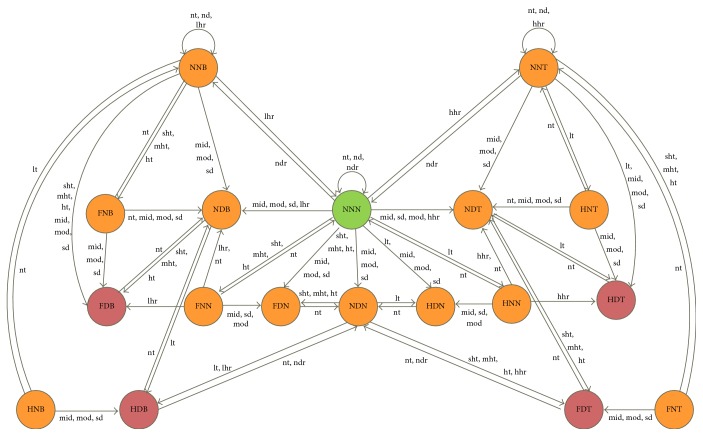
Fuzzy finite state machine (FSM) used in health prediction.

The defined fuzzy finite state machine is depicted in Figure 3, which shows all possible transition paths, health judgment rules, health symptoms, and states graphically.

#### 2.2.3. Health State Transition Metrics

In order to derive the heath state transition sequence during exercise and assess the healthy degree, it is necessary for us to calculate the state transition probabilities as well as the state probabilities [16]. For each input fuzzy symptom *s*, its state transition probability *μ*(*s*) in every time step is given by

(8) $$\begin{array}{c} \mu \left(\;s\;\right)=\underset{s\in S_{i}}{\max}\left\{\;min\left(\;d_{S_{i}}\left(\;s\;\right),w\;\right)\;\right\}, \end{array}$$

where *S*_*i*_ means the symptom set of the *i*th indicator (CT, DA, and HR), *d*_*S*_*i*__(*s*) signifies the DOM of *s*, and it can be achieved by the degree of membership in Figure 2, and *w* denotes the transition weight between the current state and the state we are transitioning to. The equation means that when a new symptom is acquired, we will look for the most plausible transition state. In general, the initial state probabilities of the health variables as well as the initial transition probabilities are assumed to be 1.0.

After the transition probability *μ*(*s*) has been computed, the state probability corresponding to each indicator is calculated as follows:

(9) $$\begin{array}{c} \mu_{i}\left(\;n\;\right)=\left\{\begin{array}{cc} \frac{\mu_{i}\left(n-1\right)+\mu \left(s\right)}{2}, & \\ \frac{\left(1-\mu_{i}\left(n-1\right)+\mu \left(s\right)\right)}{2}. & \end{array}\right. \end{array}$$

*μ*_*i*_(*n*) is the state probability of the *i*th indicator at the discrete time *n*. When the current state is unchanged, *μ*_*i*_(*n*) is calculated as the average of the previous state and the new computed probability. However, when the current state changes to a new state, the complement of the previous state probability is averaged with the transition probability to calculate *μ*_*i*_(*n*).

In order to evaluate the whole health status under the three input health indicators, we should deduct an overall probability *μ*_overall_(*n*) at the discrete time *n* for the current health state.

(10) μoveralln=1/N+1∑i=1Nμin−1+μoveralln−12+1/M∑i=1Mμin−12,

where *N* is the number of indicators that did not change, *M* is the number of indicators that did change, and *μ*_*i*_(*n* − 1) is the state probability of the *i*th indicator at time *n* − 1.

## 3. Experiments and Discussion

### 3.1. Thermophysiological Model Validation

To validate the integrated thermophysiological simulation model, five adult male subjects are selected to do exercise in two different scenes [6]. The average information of the subjects is 21.7 years, 176.8 cm, and 72.2 kg. The detailed settings of these two exercise scenes are shown in Table 3.

**Figure 4:**
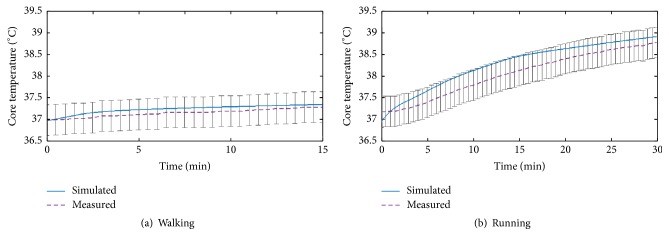
Comparison curves of core temperature.

Figure 4 shows the comparison curves of the core temperature in measurement and simulation in walking and running scenes. The pink dot line represents the measured values and the blue line represents the simulated values. The range of error bars is ±0.3°C. It can be seen that the simulated core temperature curves in both scenes have good agreements with the experimental ones and the errors between the simulated values and measured values are acceptable [43].

The weight loss before and after exercise is usually considered to be the amount of sweating. As the weight loss is easy to measure, we adopt weight loss to validate the effectiveness of sweating mechanism in our thermophysiological model.  Table 4 lists measured average weight loss and simulated water loss of the two scenes. It can be seen that the simulated values are slightly below the experimental values, and the dehydration percentages which were used to determine whether there was dehydration or not in measurement and simulation are very close.

**Figure 5:**
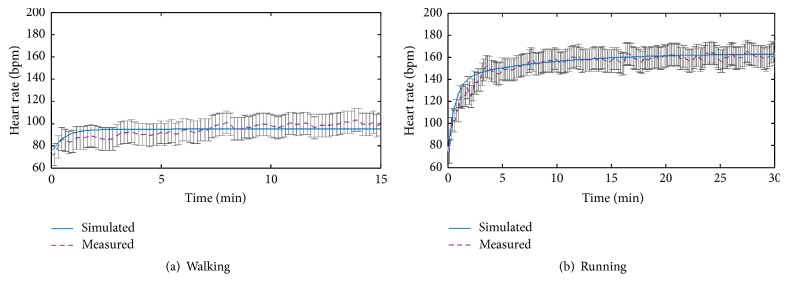
Comparison curves of heart rate.

Figure 5 shows the measured and simulated heart rate in the walking and running, respectively. The purple dot line represents the measured values and the blue line represents the simulated values. The range of error bars is ±10 bmp. In Figure 5, the heart rate values in measurement and simulation are increased sharply in the first few minutes and then slowly. The errors between the simulated values and measured values are within 10 bmp and they are acceptable [27].

Through the comparison analysis in model validation experiments, it can be concluded that the integrated thermophysiological model can well simulate physiological mechanisms as well as the dynamic changes of body physiological indicators in different ambient conditions and exercise intensities. That is, our integrated thermophysiological model is effective and it is feasible to apply this model for exercise health prediction.

### 3.2. Exercise Health Prediction Cases

After human thermophysiological model validation, two exercise health prediction cases with different subjects, clothes, external environments, and exercise intensities are designed in Table 5. The thermophysiological simulated results and the corresponding health state transition list are given and discussed.

*Case 1*. Subject A (25 yrs, male, 68 kg, and 1.77 m) participates in treadmill exercise at the speed of 7 km/h (jogging) for 2 hours while wearing shorts and a t-shirt in environment conditions of 25°C and 65% RH. The related parameters in our thermophysiological model for subject A are estimated in [27, 44] and they are set as *k*_*a*_ = 250, *k*_sw_ = 100, *a*_1_ = 1.84, *a*_2_ = 24.32, *a*_3_ = 0.0636, *a*_4_ = 0.00321, *a*_5_ = 8.32, and *a*_6_ = 0.38. Other parameters needed in exercise health prediction like body areas, THR, and MHR are calculated and their values are *A* = 19199 cm^2^, MHR = 176.05, and THR = 145.435.

**Figure 6:**
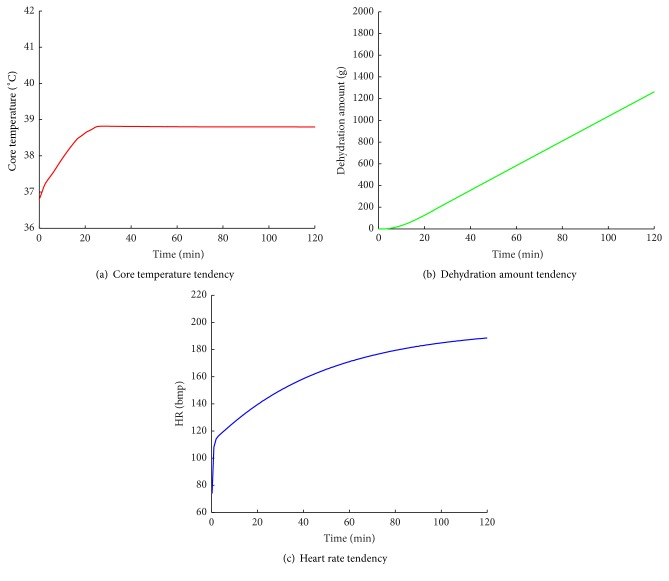
The change curves of the simulated physiological indicators of subject A.

Figure 6 shows the simulation results of the thermophysiological model. In Figure 6(a), the core temperature increases rapidly in the first twenty minutes, and then it remains approximately 38.8°C. At the same time, lots of sweat are secreted; the changes of sweat accumulation (dehydration amount) are shown in Figure 6(b).  Figure 6(c) shows the change curve of the simulated heart rate.

By analyzing the simulated indicator values, a series of fuzzy symptoms along with their probabilities are extracted and the assessed health states are listed in Table 6. In Table 6, the values behind the states are the probabilities of the current state. While a new set of fuzzy symptoms is extracted, the current state probabilities can be updated by (10). The greater the probability value, the greater the likelihood for the subject to be in the current state. For example, from the 6th minute to the 10th minute, the probability of FNN is increased from 0.68 to 0.98. That is to say, while the core temperature increases and reaches slightly high temperature, the body health state of the subject A is in FNN with a high-probability. As listed in Table 6, the user is initially in state NNN and its corresponding probability is 1. The end state is FDT and its corresponding probability is 0.89. In the 5th minute of simulation, the fuzzy symptom of core temperature changes from nt to dt and the health state changes from NNN to FNN; at this time, subject A's core temperature is higher than normal body temperature. As the core temperature continues to increase, the fuzzy symptom of core temperature changes into mt and lasts until the end of simulation. With the heart rate increasing during running, the fuzzy symptom of HR is from nhr to hhr at the 49th minute. The health state is from FNN to FNT correspondingly. Besides, people are dehydrated at the 114th minute, and the fuzzy symptom of DA is from nh to mih and the health state is from FNT to FDT.

It is known that high body temperature in people for a long time is harmful to the human organs and physiological functions. Some symptoms like dehydration and heatstroke usually appear at the same time. Hence, when the current state is FNN, especially when the fuzzy symptom of CT is mht, health warning should be given to users and heat dissipation of body should be enhanced. While the fuzzy symptom of DA is mih, people must drink more water to stay hydrated and to stay in a good physiological condition. Moreover, tachycardia for a long time also can cause poor physical fitness. We should adjust the sport plans while the symptom hhr of HR arises.

In short, during the whole simulation process, the body goes through four states: NNN, FNN, FNT, and FDT. This health state tendency agrees with the real physiological changes. Based on the simulated results, we can take reasonable behaviors to avoid potential health risk.

*Case 2*. Subject B (35 yrs, male, 74 kg, 1.73 m) participates in treadmill exercise at the speed of 12 km/h (running) for 0.5 hours while wearing shorts and a vest in environment conditions of 28°C and 50% RH. The related parameters in our heat physiological models for subject B are set as follows: *k*_*a*_ = 50, *k*_sw_ = 10, *a*_1_ = 2.2, *a*_2_ = 19.96, *a*_3_ = 0.0831, *a*_4_ = 0.002526, *a*_5_ = 8.32, *a*_6_ = 0.38, *A* = 19697 cm^2^, MHR = 181.55, and THR = 160.04 [28, 35].

**Figure 7:**
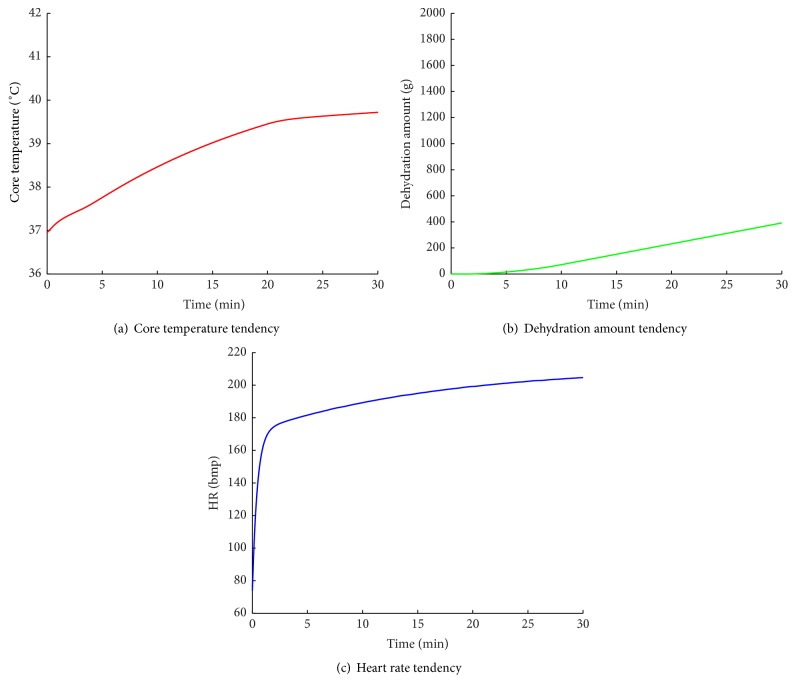
The change curves of the simulated physiological indicators of subject B.

The tendency curves of core temperature, dehydration amount, and heart rate of subject B are shown in Figure 7. Compared with jogging of subject A, the physiological values of subject B increase more quickly. Particularly the core temperature increases to 40°C and the heart rate increases to 170 immediately. The corresponding health state transition sequence is shown in Table 7.

In Table 7, the human health symptom goes through three states: NNN, NNT, and FNT. At the 2nd minute, the fuzzy symptom of HR changes from nhr to hhr and the current health state changes from NNN to NNT. And immediately following that, the fuzzy symptom of core temperature changes from nt to sht, mht, and ht; the health state changes in FNT. During this case, the sweat accumulation is in the normal range; its related symptom is nh during the whole simulation process. As the heart rate sharply increased to the MHR, the exercise performed by subject B is risky. That is, subject B is not suitable for this running plan. We should adjust the running intensity or running time.

### 3.3. Discussion

The experiments show that our approach can simulate the physiological changes of human body and predict the health states in different exercises. Furthermore, important exercise health warnings can be given to participants when the human body gets into a risky health state [45]. This is very helpful for individual when he (or she) is not sure about how long he (or she) should be running in a specific environment temperature while maintaining a healthy state. And the appropriate exercise suggestions also can be given according to the simulated health states before they start the exercise.

Case 1 shows that jogging for a long time may cause a mild dehydration phenomenon, although in a pleasant environment. This is because sweating takes effect in thermoregulation system and a lot of sweat is secreted in the whole exercise process. So water should be supplemented in a longtime jogging in time and exercise duration should be arranged reasonably (e.g., not more than 2 hours) [44, 46].

Case 2 simulates the physiological changes of human body in fast running. When fast running is more than 15 minutes in an environment temperature of 28°C, human body reaches a high load state (performance at core temperature and heart rate). Therefore, our simulation result suggests that fast running should not be more than 15 minutes when the environment temperature exceeds 28°C [47, 48]. Also, a fast running is not suitable for the people with heart disease, since the heart rate sharply increases at the first minutes of running.

## 4. Conclusion

During exercise, the physiological changes of human body are caused by the various physiological regulation mechanisms such as thermoregulation and cardiovascular regulation. These physiological mechanisms are directly related to the health evaluation and prediction. For the purpose of obtaining the human exercise health, we propose a novel exercise health simulation approach, which comprises an integrated thermophysiological model and a fuzzy finite state machine. Some common physiological indicators like core temperature, dehydration amount, and heart rate used in exercise health prognosis can be well simulated by our thermophysiological model. Then a fuzzy finite state machine is defined to describe the health state transition during exercise, and the health status can be obtained at an earlier stage.

The further work is discussed as follows: (1) The exercise health simulation and analysis in this paper are aimed at healthy people; the similar research on specific populations (such as cardiac patients or other unhealthy people) should be analyzed and discussed; (2) the real-time exercise monitoring is a hot research topic. We have proposed a real-time exercise monitoring framework based on the given thermophysiological model; its corresponding real-time exercise monitoring APP has been implemented. However, with the increasing of client users, the problems such as simulation efficiency, load balancing, and the analysis and storage of the increasing physiological data are yet to be solved.

## Figures and Tables

**Table 1:** Notations in FSM.

Health variables	States	Symptoms
Core temperature (CT)	H (hypothermia)	lt (low temperature)
	N (normothermia)	nt (normal temperature)
	F (hyperthermia)	sht (slightly high temperature)
		mht (moderate high temperature)
		ht (high temperature)

Dehydration amount (DA)	N (normal)	nd (nondehydration)
	D (dehydration)	mid (mild dehydration)
		mod (moderate dehydration)
		sd (severe dehydration)

Heart rate (HR)	B (bradycardia)	lhr (low heart rate)
	N (normal)	nhr (normal heart rate)
	T (tachycardia)	hhr (high heart rate)

**Table 2:** Health states and syndromes in FSM.

Health state (CT∖DA∖HR)	Syndrome (possible)
HNB	Hypothermia, arrhythmia
HNN	Hypothermia
HNT	Hypothermia, arrhythmia
HDB	Hypothermia, dehydration, arrhythmia
HDN	Hypothermia, dehydration
HDT	Hypothermia, dehydration, arrhythmia
NNB	Bradycardia
NNN	Normal
NNT	Tachycardia
NDB	Dehydration, arrhythmia
NDN	Dehydration
NDT	Dehydration, arrhythmia
FNB	Hyperthermia, arrhythmia
FNN	Hyperthermia
FNT	Hyperthermia, arrhythmia
FDB	Hyperthermia, dehydration, heatstroke, syncope, arrhythmia
FDN	Hyperthermia, dehydration, heatstroke, syncope
FDT	Hyperthermia, heatstroke, syncope, arrhythmia, shock

**Table 3:** Scene settings for the model validation.

Scene	Environment conditions	Exercise settings
1	25°C, 70% RH	Walking at 5 km/h for 15 min
2	30°C, 50% RH	Running at 8 km/h for 30 min

**Table 4:** Comparison values of water loss.

	Measured results		Simulated results	
	Water loss (g)	Dehydration percentage^1^	Water loss (g)	Dehydration percentage^1^
Walking	41	0.057%	30.98	0.043%
Running	505	0.699%	419.35	0.581%

^1^Dehydration percentage: the ratio between water loss and body weight.

**Table 5:** Case settings and parameters.

Case	Settings										
	Subject setting			Clothing setting		Environment Setting		Exercise Setting			
	Age (years)	Height (cm)	Weight (kg)	Material	Coverage rate	Temperature (°C)	Relative humidity	Exercise type	Speed (km/h)	EIP	Duration (min)
Case 1	25	177	68	Cotton	70%	25	65%	Jogging	7	60%	120
Case 2	35	173	74	Cotton	50%	28	50%	Running	12	80%	30

	Parameters										
	*k*_*a*_		*k*_sw_	*a*_1_	*a*_2_	*a*_3_	*a*_4_	*a*_5_		*a*_6_	

Case 1	250		100	1.84	24.32	0.0636	0.00321	8.32		0.38	
Case 2	50		10	2.2	19.96	0.0831	0.002526	8.32		0.38	

**Table 6:** Health state transition sequence of subject B.

Time (min)	Current state & Prob^1^	CSCT & Prob^2^	CSD & Prob^3^	CSHR & Prob^4^
*t* = 0	NNN 1	nt 1	nd 1	nhr 1
*t* = 1	NNN 1	nt 1	nd 1	nhr 1
*t* = 2	NNN 1	nt 1	nd 1	nhr 1
*t* = 3	NNN 1	nt 1	nd 1	nhr 1
*t* = 4	NNN 1	nt 0.7	nd 1	nhr 1
*t* = 5	NNN 0.95	nt 0.73	nd 1	nhr 1
*t* = 6	FNN 0.68	sht 1	nd 1	nhr 1
*t* = 7	FNN 0.84	sht 1	nd 1	nhr 1
*t* = 8	FNN 0.92	sht 1	nd 1	nhr 1
*t* = 9	FNN 0.96	sht 1	nd 1	nhr 1
*t* = 10	FNN 0.98	sht 0.9	nd 1	nhr 1
*t* = 11	FNN 0.97	mht 0.56	nd 1	nhr 1
*t* = 12	FNN 0.91	mht 1	nd 1	nhr 1
*t* = 13	FNN 0.96	mht 1	nd 1	nhr 1
⋮				
*t* = 47	FNN 0.86	mht 1	nd 1	nhr 0.54
*t* = 48	FNN 0.85	mht 1	nd 1	nhr 0.52
*t* = 49	FNN 0.85	mht 1	nd 1	hhr 0.5
*t* = 50	FNT 0.61	mht 1	nd 1	hhr 0.52
*t* = 51	FNT 0.73	mht 1	nd 1	hhr 0.54
⋮				
*t* = 113	FNT 0.86	mht 1	nd 0.52	hhr 1
*t* = 114	FNT 0.85	mht 1	mid 0.51	hhr 1
*t* = 115	FDT 0.62	mht 1	mid 0.55	hhr 1
*t* = 116	FDT 0.73	mht 1	mid 0.58	hhr 1
*t* = 117	FDT 0.8	mht 1	mid 0.61	hhr 1
*t* = 118	FDT 0.83	mht 1	mid 0.65	hhr 1
*t* = 119	FDT 0.86	mht 1	mid 0.68	hhr 1
*t* = 120	FDT 0.88	mht 1	mid 0.71	hhr 1
—	FDT 0.89	—	—	—

^1^Prob: probability; ^2^CSCT: current symptoms of core temperature; ^3^CSD: current symptoms of dehydration; ^4^CSHR: current symptoms of heart rate.

**Table 7:** Health state transition sequence of subject B.

Time (min)	Current state & Prob^1^	CSCT & Prob^2^	CSD & Prob^3^	CSHR & Prob^4^
*t* = 0	NNN 1	nt 1	nd 1	nhr 1
*t* = 0.5	NNN 1	nt 1	nd 1	nhr 1
*t* = 1	NNN 1	nt 1	nd 1	nhr 0.86
*t* = 1.5	NNN 0.98	nt 1	nd 1	nhr 0.52
*t* = 2	NNT 0.63	nt 0.73	nd 1	hhr 0.68
*t* = 2.5	NNT 0.72	sht 0.58	nd 1	hhr 0.76
*t* = 3	FNT 0.6	sht 0.87	nd 1	hhr 0.82
⋮				
*t* = 6	FNT 0.99	sht 0.94	nd 1	hhr 1
*t* = 6.5	FNT 0.98	sht 0.56	nd 1	hhr 1
*t* = 7	FNT 0.92	mht 0.8	nd 1	hhr 1
*t* = 7.5	FNT 0.93	mht 1	nd 1	hhr 1
⋮				
*t* = 13.5	FNT 1	mht 1	nd 1	hhr 1
*t* = 14	FNT 1	mht 0.9	nd 1	hhr 1
*t* = 14.5	FNT 0.98	mht 0.65	nd 1	hhr 1
*t* = 15	FNT 0.93	ht 0.6	nd 1	hhr 1
*t* = 15.5	FNT 0.9	ht 0.84	nd 1	hhr 1
*t* = 16	FNT 0.92	ht 1	nd 1	hhr 1
⋮				
*t* = 29.5	FNT 1	ht 1	nd 1	hhr 1
—	FNT 1	—	—	—

^1^Prob: probability; ^2^CSCT: current symptoms of core temperature; ^3^CSD: current symptoms of dehydration; ^4^CSHR: current symptoms of heart rate.
